# Assessing barriers and opportunities for the improvement of laboratory performance and robust surveillance of antimicrobial resistance in Nigeria– a quantitative study

**DOI:** 10.1186/s13756-025-01530-9

**Published:** 2025-04-12

**Authors:** Obiageli J. Okolie, Sanda U. Ismail, Uzoma Igwe, Emmanuel C. Adukwu

**Affiliations:** 1 School of Applied Sciences, University of the West England, Bristol, UK; 2 School of Health and Social Wellbeing, University of the West England, Bristol, UK

**Keywords:** Antimicrobial resistance, Laboratory systems, Laboratory networks, AMR surveillance, Nigeria

## Abstract

**Background:**

Good quality data is essential in optimising containment strategies for antimicrobial resistance, a global public health threat estimated to cause around 10 million deaths yearly and up-to 5% loss in GDP by 2050 if left unaddressed. The laboratory system plays an important role in the collection of high-quality data as well as ensuring validity, reliability and timeliness of data. However, in many low-medium income countries including Nigeria, the technical capacity of the laboratory for fulfilling these responsibilities is unknown. This paucity of information limits piloting of strategies to complement existing surveillance and planning improvement of subsequent laboratory iterations into the surveillance system. The focus of this study was to assess the gaps, vulnerabilities and enablers of laboratory strengthening processes in the scope of technical capacity for clinical and public health functions and to provide a roadmap for improved surveillance of antimicrobial resistance in Nigeria.

**Methods:**

A cross-sectional study design utilising structured questionnaire administered online via Qualtrics and reported in accordance with strengthening the Reporting of Observational Studies in Epidemiology (STROBE) guidelines. Data analysis involved descriptive and inferential statistics as well as bivariate and multivariate logistics to test predictive analysis of relationship between variables.

**Results:**

A total of 302 laboratories completed the questionnaire, 107 (53.4%) government laboratories and 195 (64.6%) private sector laboratories. 18.2% reported excellent knowledge, 25.5% has excellent capacity, 7.3% are fully ready for surveillance, 12.3% are participating in some surveillance, and 1.0% record important microbiological data that correlates with epidemiological information.

**Conclusion:**

Tertiary laboratories reported highest performance across all surveillance quality indicators (SQIs). AMR surveillance is skewed toward government and tertiary laboratories, leaving lower-level and rural facilities underutilized despite their potential. This results in missing community-level data and undermines the representativeness of surveillance. The study identifies gaps in recruitment, assessment, and oversight but also offers strategies to address these issues.

**Supplementary Information:**

The online version contains supplementary material available at 10.1186/s13756-025-01530-9.

## Background

Antimicrobial resistance (AMR) is regarded as one of the most important public health threats and has been attributed to significant mortality and morbidity worldwide. According to the Antimicrobial Resistance Collaborators [1], AMR was directly responsible for 1.27 million global deaths in 2019 and contributed to 4.95 million deaths. While there are several efforts at national and international level to strengthen AMR surveillance systems e.g. Global Antimicrobial Resistance and Use Surveillance System (GLASS) etc. and progress with National Action Plans, (NAPs), current reports still show that in low and middle income countries, these efforts remain slow-paced and may not be fully implemented due to factors such as inadequate resources, political will, supply chain issues, inadequate training and among other things, poor healthcare and laboratory infrastructure [2, 3].

Laboratory networks are a core component of all surveillance and health systems [4]. Accurate and timely laboratory information is at the centre of the efficient treatment, management and prevention of infectious and non-infectious diseases [5]. Globally, public health interventions/policies largely rely on data from laboratories and particularly, in times of serious public health crisis, laboratories are at the heart of investigation and response procedures [6]. The difficulties encountered in providing timely laboratory testing during epidemic and pandemics highlight that global health security relies on adequate public health laboratory capacity in all regions [7].

The role of laboratory services and the efforts to strengthen them, for both clinical and public health functions are increasingly recognised [8, 9]. Given the important role of the laboratory in antimicrobial resistance (AMR) surveillance and response mechanisms, laboratory assessment in-line with surveillance objectives of the National Action Plan for Antimicrobial Resistance (NAPAR) is essential for strengthening laboratory capacity and planning subsequent laboratory iterations into the surveillance system. More so, laboratory assessment provides invaluable indicators that can be used to gauge the level of surveillance implementation [10]. This assessment should consider all aspects of AMR surveillance activities including: clinical sampling, laboratory testing procedures, specimen sharing, reagents and equipment supplying systems, data management, human resourcing, and infrastructure [11, 12]. Assessing the organisational structure of the laboratories at local, regional and national levels is also crucial as it impacts the functionality of the laboratory and flow of surveillance data across time and space [11, 13].

In Nigeria, the 2017 National Action Plan (NAP) for AMR highlighted that strengthening knowledge and evidence through surveillance is a strategic priority for tackling AMR. In actualising this NAP priority, the laboratory plays a central role not only for detecting, confirming, and reporting resistant pathogen to the surveillance network, but also supporting global, regional and local containment efforts through provision of other useful information that guides policy action and clinical trials [6]. To inform meaningful action, the laboratory as part of the surveillance eco-system must guarantee timely, valid, and reliable data as well as correlation of this data with important demographic information but the extent to which the laboratories fulfil these technical responsibilities is not clear [14]. Specifically, in relation to AMR, a balanced geographical and socio-economic distribution of surveillance sites is essential for robust surveillance and further assures that the generated data is representative of the population under surveillance, inclusive and not skewed [15].

The status of the laboratory networks for AMR surveillance in Nigeria has not been assessed post-NAP implementation. What remains unclear is if the technical capacity, organisational structure, and hierarchical criteria followed in the recruitment of laboratories, as well as the distribution of laboratories across geographical settings, aligns with surveillance system requirements and protocols. This cross-sectional study will fill these knowledge gaps and thus, provide a snapshot of representative samples of laboratories at various levels in Nigeria which is useful for assessing surveillance system performance and to provide a roadmap for equitable laboratory recruitment which is vital for expansion of laboratory networks and achieving comprehensive surveillance.

## Methodology

### Study design

This cross-sectional study was conducted between December 2020 and July 2021 among government and private laboratories operating at all healthcare levels in Nigeria and was reported in accordance with Strengthening the Reporting of Observational Studies in Epidemiology (STROBE) guidelines [16].

### Study population and setting

**Fig. 1:**
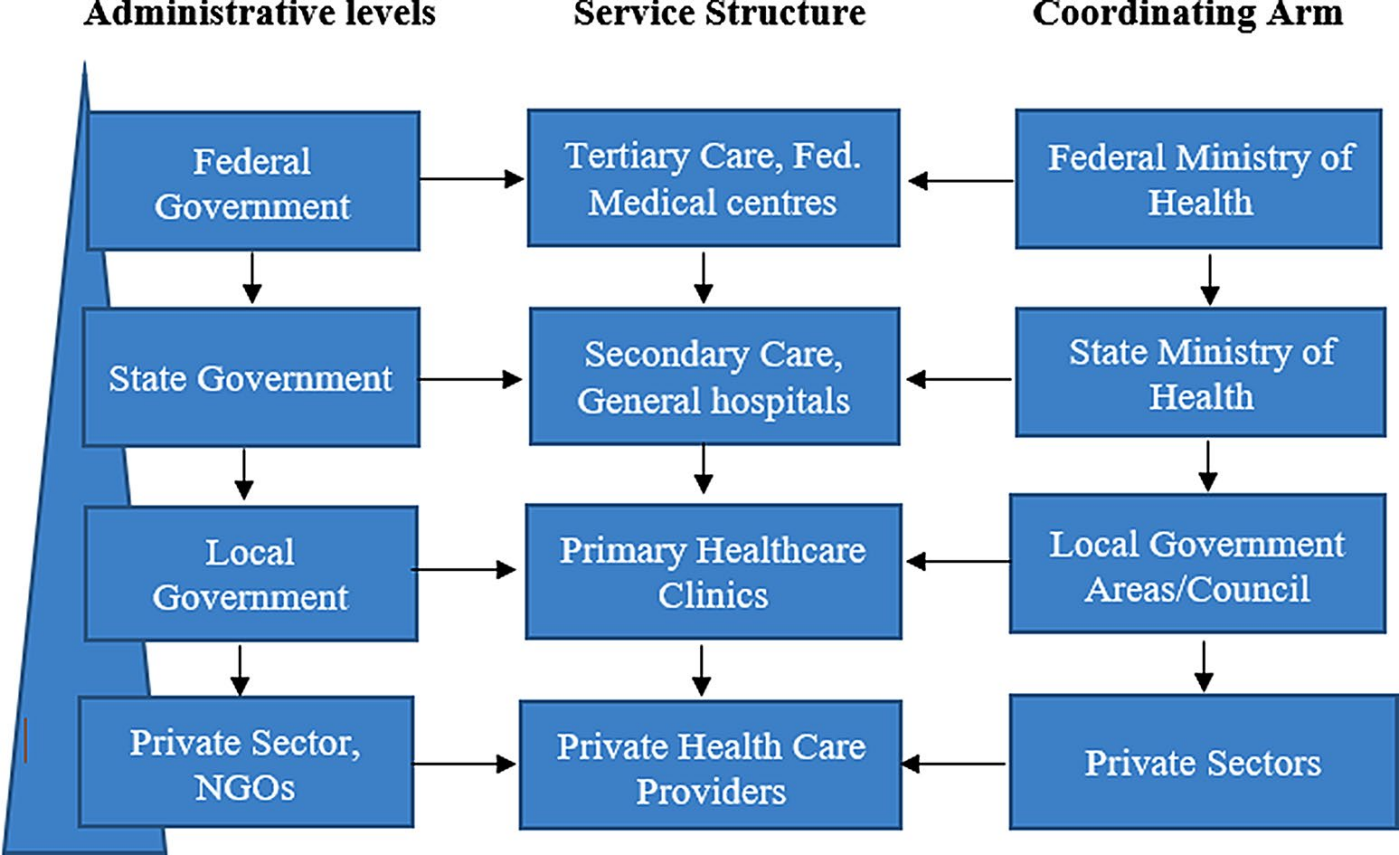
Hierarchical organisation of healthcare system and governance structure in Nigeria (a top-bottom approach)

The setting for this study is Nigeria, a federated system of government consisting of a Federal Government, 36 State Governments, the Federal Capital Territory (FCT) and 774 Local Government Areas. The states and the FCT are further grouped into six geo-political zones: South-South, South-East, South-West, North-East, North-West, and North-Central [17]. Nigeria operates a tiered system of government and a hierarchical healthcare system (Primary, Secondary and Tertiary) encompassing federal and state-owned facilities (Fig. 1). The laboratory network spans across different levels of healthcare and can be categorised by: ownership (private, government), tiers (tertiary, secondary and primary), affiliation (teaching hospitals, state medical centres, primary health centres, federal ministries and port health), dependent (laboratories connected to healthcare facility), and independent (laboratories not connected to healthcare facility).

### Study participants

In selecting the study participants and to minimise sampling bias, we defined a target population (using predetermined eligibility criteria) and sampling frame with the list of groups that the sample will be drawn from to ensure inclusion of participants from each geo-political zone. To be eligible, the organisation must either be a professional body consisting registered microbiologists, laboratory scientists, technicians/assistants and epidemiologists; a collection of laboratory scientist in private (non-government) practice and comprises directors/managers or a body responsible for regulatory activities across private and government establishments. In a bid to generate representative data, and to allow for sub-group analysis, we adopted a purposive sampling technique to guarantee recruitment of laboratories to span private and government, tertiary, secondary and primary care laboratories as well as laboratories in urban and rural settings. Three professional bodies were identified that fit the sampling frame; Association of Medical Laboratory Scientists of Nigeria, Guild of Medical Laboratory Directors (GMLD) and Medical Laboratory Science Council of Nigeria (MLSCN). Thus, all potential respondents within the sample frame had equal chance of participating in the survey. An initial request letter to participate in the study with detailed study information including participant information sheet was emailed to the representative of each of the three professional bodies to solicit their member participation. This was followed up with telephone calls and further correspondence with the contact persons before an agreement to participate was received. Following the receipt of clearance, a unique survey link was generated and sent to the respective contact persons of the professional bodies who distributed same to their members via their internal membership email list.

### Sample size

Sample size for this study was determined using Event Per Variable (EPV) based on the study objective, the statistical analysis involved, and the type of variables involved [18]. EPV utilises the number of event-per-predictor variable in a study to determine the ideal sample size. For observational studies with large population size that involve logistic regression, the EPV of 50 rule is advised in order to achieve significant sample size and high statistical power [19]. An EPV of 50 was adopted which yields a larger sample size, thereby minimising the risk of type II error associated with small sample size. Substituting values into the EPV formulae [EPV = 100 + (x) (i)] yields a sample size of 300. Where (x) is an integer chosen by the researcher, (i) is the number of independent variables and 100 is constant;

EPV = 100 + (50) x [4] i.e. {100 + 200 = 300}.

Therefore, the minimum sample size required to achieve significant power for this study is 300.

### Data collection instruments and measurements

A structured standardised self-administered and pretested questionnaire was distributed to the study participants online via Qualtrics^XM^ software [20]. The questionnaire comprised of 49 questions divided into five Surveillance Quality Indicators (SQIs) sections: (a) knowledge of AMR surveillance (b) laboratory capacity for AMR surveillance (c) readiness to participate in AMR surveillance (d) status of AMR surveillance participation (e) capturing of important AMR surveillance data. The questionnaire also captured demographic variables: laboratory location by State; catchment area of laboratory; laboratory affiliation (government, private); laboratory connection (tertiary, secondary, and primary); major source of samples; and respondent’s occupational role.

The survey questionnaire was adapted from the WHO questionnaire for assessment of national networks for AMR surveillance and questionnaire for assessing laboratory capacity [21]. The questionnaire was further validated by three experts in the fields of laboratory assessment and public health to ensure it contained questions that cover all aspects of the construct being measured. The validation process was in two phases: firstly, the full domain of content relevant to the study was defined; in the second phase, specific areas from the domain relevant to the study objectives were sampled and tested to establish that the items are representative of the intended study outcome. The content validity index individual (I-CVI) was utilised to evaluate the relevance of individual item on a 4-Likert scale (from 1 = non-relevant to 4 = very relevant). Then for each question, the number of participants giving 3 or 4 score (relevant) against those giving 1 or 2 (non-relevant) were counted and the proportion was calculated. All individual participants’ scoring was in agreement with the order, hence l-CVI score of 1.0 was assigned. This score aligns with [22], that where five or fewer experts are involved, all must agree (i.e. l-CVI of 1.0) to overcome problem of chance agreement. To further give inference on comprehensiveness of the whole questionnaire, content validity index scale (S-CVI) was used to evaluate the validity of the overall scale. Again, the S-CVA validation shows a universal agreement from all experts. A pre-test of the questionnaire was conducted among 15 randomly selected laboratories to assess ease of administration. Based on the feedback received, the questions were further modified to ease understanding.

### Statistical method

The collected data were checked for completeness and errors before analysis. Incomplete questionnaires were excluded and counted as a non-response. All fully completed questionnaire were entered into Microsoft Excel and exported to SPSS version 20 for analysis. A p-value cut off of 0.05 was used to determine the level of statistical significance.

Study variables were summarised using frequencies and mean. A Chi-square test was used to describe the strength and significance of relationship between the SQI (dependent) and the laboratory demographics (independent), binomial and multinomial logistic regression were then used to determine associations between the two variables. Bivariate correlation was used to check for linear relationship (correlation coefficients) and the direction of the relationship between demographic variables and the SQI to determine if the impact of a change in one variable will have a significant impact on the other. The correlation coefficients were interpreted in accordance to [23] guideline.

The survey responses were scored as valid for eligible response, or invalid where missing, or ‘‘do not know’’ answers were entered. A score of one [1] was assigned for every valid response and zero (0) was given to invalid responses in accordance with [24] scoring approach. The score for each of the five SQIs per respondent was presented as percentage of the maximum possible score for each indicator using this calculation (score obtained/total possible score × 100%) as earlier documented by [21]. The ranking used for knowledge followed a previously adopted categorisation by [25, 26] who ranked knowledge theme scores of ≤ 49% as poor, 50–75% as moderate and ≥ 80 as excellent. The ranking for laboratory capacity followed [27] ranking which assigned a score of ≤ 59% as weak capacity, 60–80% as good capacity, and > 80% as strong capacity. The rest of the SQI scores were assigned relative to features of the data and not based on pre-established ranking categorisation. For status of surveillance participation, a score of ≤ 59% was regarded as poor participation, 60–80% was regarded as fair participation, while > 80% was taken as good participation. For readiness to partake in AMR surveillance, a score of ≤ 59% was regarded as not ready, 60–80% was taken as fairly ready, while > 80% was regarded as fully ready. On the AMR surveillance data capturing, a score of ≤ 59% was regarded as not capturing important AMR surveillance data, 60–89% was taken as partially capturing important AMR surveillance data, while a score of ≥ 90% was taken as fully capturing AMR surveillance data. In addition, the scores for each SQIs were further categorised into poor, moderate/fair and good/excellent scores.

## Results

### Sociodemographic data

**Fig. 2:**
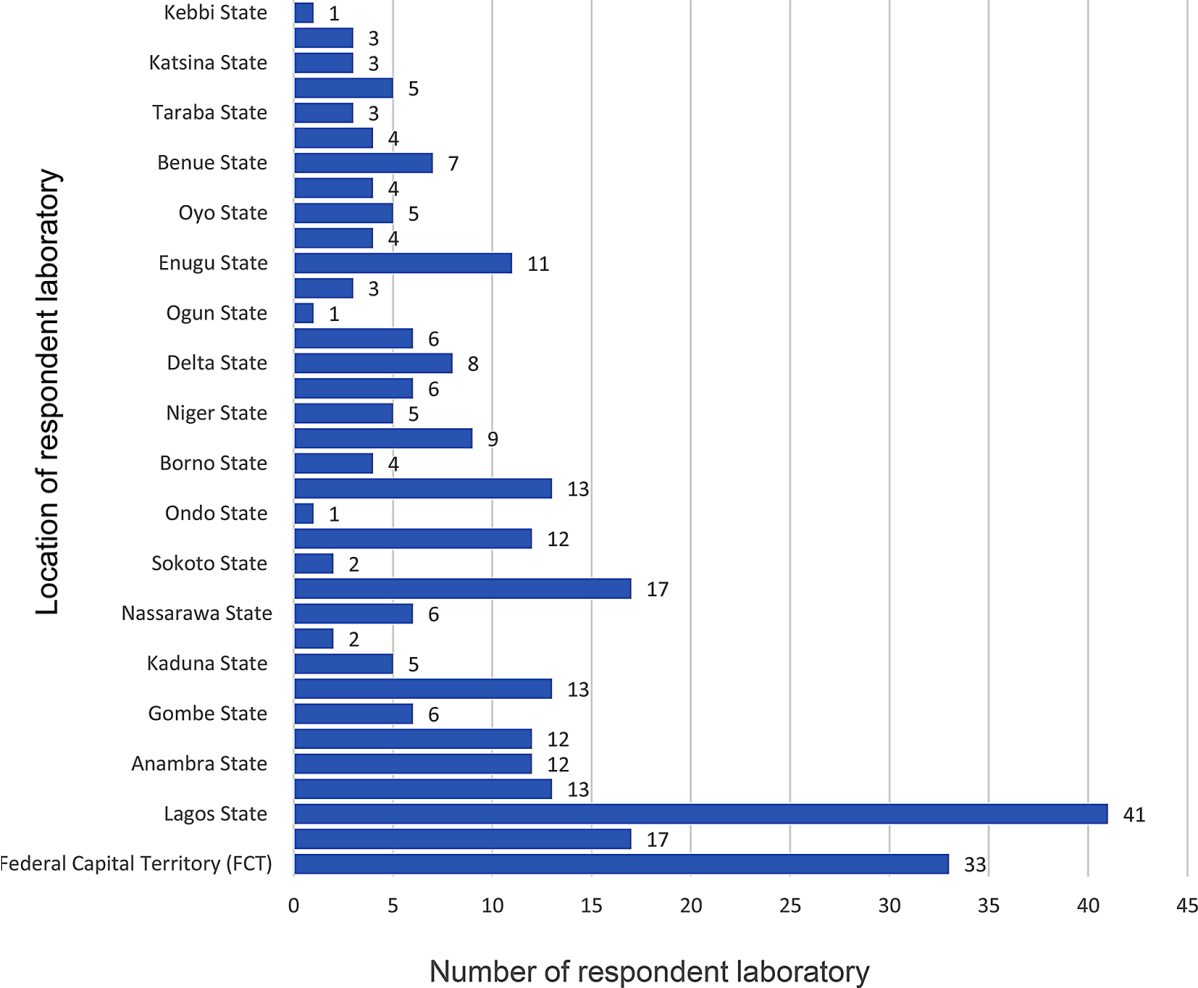
Distribution of respondent laboratory by State

A total of 310 laboratories responded to the survey. The survey was completed by directors, practice managers, senior staff members or a decision maker in the organisation to ensure reliability, accuracy and authenticity of information. Only 302 (97.4%) completed responses were analysed; eight (2.6%) had incomplete information and were excluded. The demographic characteristics of the respondents is available in shown in Table 1. Of the 302 complete responses, 107 (53.4%) responses were from laboratories with government affiliation, while a higher response of 195 (64.6%) was recorded from laboratories with private affiliation. Based on laboratory connection, independent laboratories accounted for 123 (40.7%) responses, while laboratories connected/linked to teaching hospitals, federal medical centres, general/district hospitals, primary healthcare and private hospitals accounted for 37 (12.3%), 26 (8.6%), 44 (14.6%), 12 (4.0%) and 60 (19.4%) responses respectively. Geopolitically, data showed that highest response was recorded from laboratories located in the South-South zone 72 (23.8%), while South-West, South-East, North-Central, North-West and North-East accounted for 53 (17.5%), 66 (21.9%), 64 (21.2%), 24 (7.9%) and 23 (7.6%) responses respectively. Majority of the laboratories 283 (93.7%) reported their source of sample is from humans, 7 (2.3%) reported sample source from animals, 5 (1.7%) reported sample source from environment, only 7 (2.3%) reported sample source across human, animal and environment (Table 1). One hundred and twenty-eight (128) laboratories reported to follow international quality standards; 95 (35.1%) utilise CLSI, 22 (7.3%) utilise EUCAST, 4 (1.3%) utilise BSAC and 7 (2.3%) utilise textbook. Laboratories were also grouped according to their indicated state of operation as shown in Fig. 2.

**Table 1:** Demographic characteristics of respondent laboratories

Laboratory Ownership	*n* (%)
Government Owned	107 (35.4)
Private Owned	195 (64.6)
**Laboratory Affiliation**	n (%)
Teaching Hospital	37 (12.3)
Federal Medical Centre	26 (8.6)
General/District Hospital	44 (14.6)
Primary Health Care	12 (4.0)
Private Hospitals	60 (19.4)
Independent Laboratory	123 (40.7)
**Geopolitical Zones**	n (%)
South-South	72 (23.8)
South-West	53 (17.5)
South-East	66 (21.9)
North-Central	64 (21.2)
North-West	24 (7.9)
North-East	23 (7.6)
**Sources of Samples**	n (%)
Human samples	283 (93.7)
Animal samples	7 (2.3)
Environmental samples	5 (1.7)
All Samples Sources	7 (2.3)

Key: n=number, %=percentage

### Distribution of surveillance quality indicator scoring

#### Knowledge

**Fig. 3:**
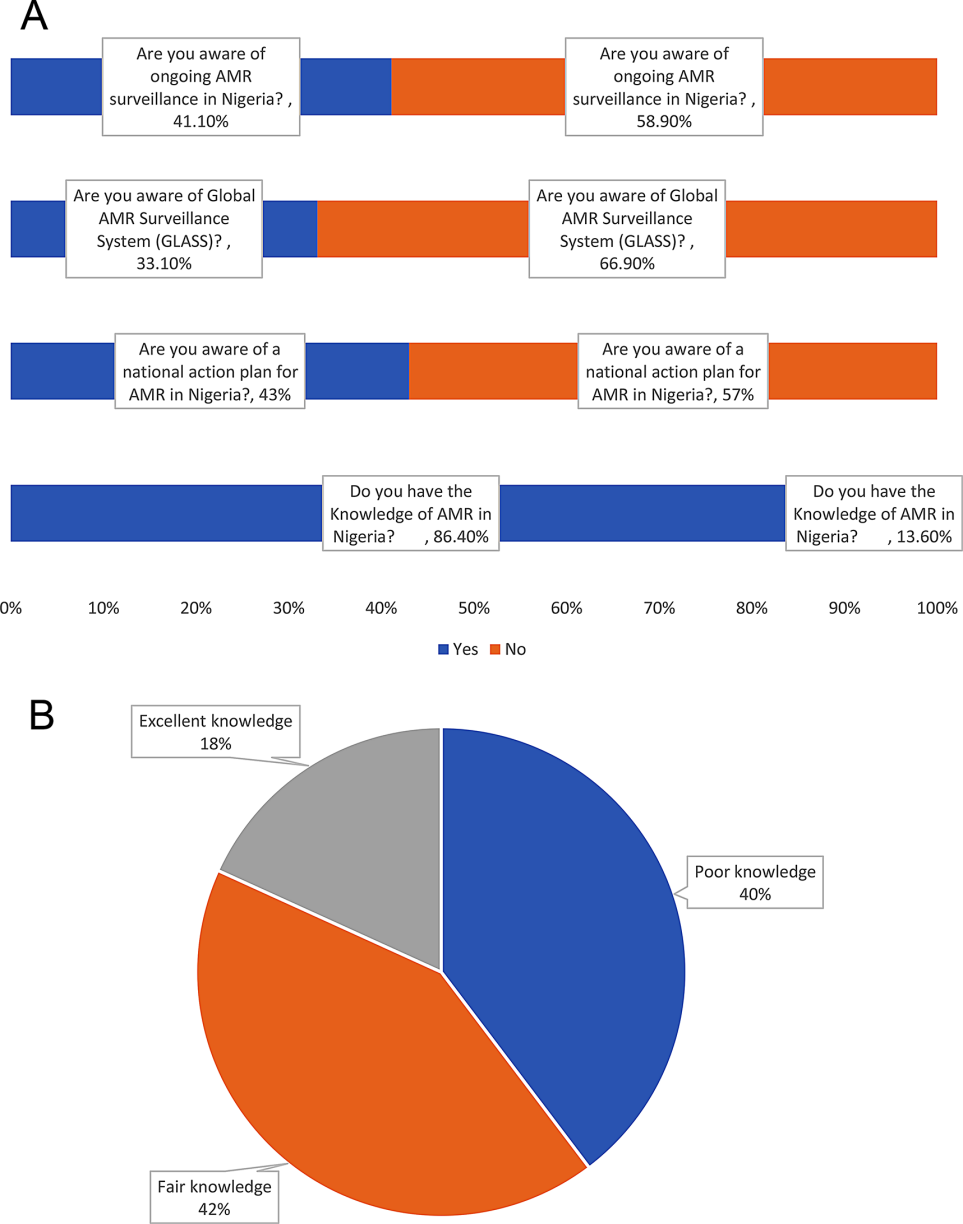
**a**) Responses to knowledge questions; with average knowledge score = 50.99 ± 32.79%. **b**) Percentage distribution of knowledge score (*n*=302)

The knowledge indicator had four questions. Of the 302 complete responses, 261 (86.4%) reported knowledge of AMR surveillance, 130 (43.0%) reported knowledge of the national action plan for AMR, 124 (41.1%) reported knowledge of ongoing AMR surveillance in Nigeria, and 100 (33.1%) had knowledge of the Global AMR and Use Surveillance System (GLASS); data shown in Fig. 3a. The mean score for knowledge was 50.99%: 120 (39.7%) respondents had poor knowledge of AMR surveillance, 127 (42.1%) had fair knowledge, only 55 (18.2%) had excellent knowledge of AMR surveillance (Fig. 3b).

### Laboratory capacity

**Fig. 4:**
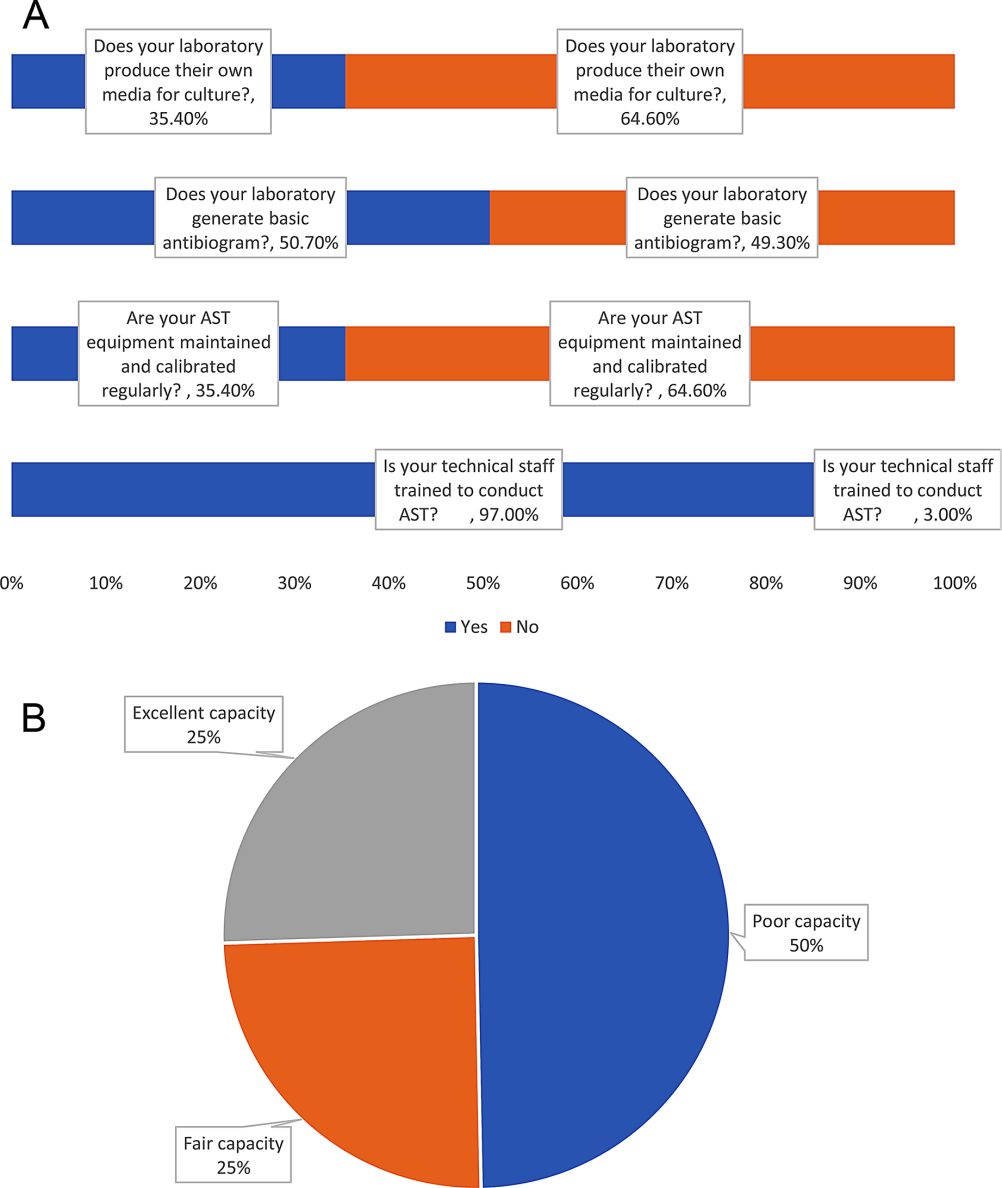
**a**) Responses to capacity questions; with average knowledge score = 58.08 ± 29.71. **b**) Percentage distribution of capacity score. (*n*=302)

The laboratory capacity indicator was comprised of five questions. Two hundred and ninety-three (97.0%) reported their technical staff were trained to conduct Antimicrobial Susceptibility Testing (AST); Fig. 4a. In addition, two hundred and seventeen, 217 (71.9%) reported the highest level of trained technical staff performing AST have a degree qualification in microbiology, 85 (28.1%) reported having a diploma, 107 (35.4%) reported regular equipment maintenance and calibration, 153 (50.7%) laboratories generate a basic antibiogram table for recording profiles of specific pathogens’ susceptibility to routinely tested antimicrobial agents and 107 (35.4%) produce their culture testing media in-house. The mean score for laboratory capacity for AMR surveillance was 58.1%: 150 (49.7%) laboratories reported poor capacity for surveillance, 75 (24.8%) reported fair capacity, and 77 (25.5%) reported good capacity for AMR surveillance (Fig. 4b).

### Laboratory readiness

The laboratory readiness indicator is comprised of ten questions. From the response received, 253 (83.8%) perform AST at their laboratory, 29 (59.2%) forward sample to other laboratories for AST, 20 (40.8%) laboratories do not report AST as reported in Table 2. Thirteen (4.3%) use manual method (agar dilution and broth microdilution), 136 (45.0%) use both disk diffusion and broth dilution, 56 (18.5%) use disk diffusion and E-test, 25 (8.3%) use both manual and automated methods, 29 (9.6%) use manual and molecular techniques, 23 (7.6%) use broth dilution. Only 128 (42.4%) laboratories reported using AST reporting guidelines. Of this number, 95 (31.5%) use Clinical and Laboratory Standards Institute (CLSI) guidelines, 22 (7.3%), 4 (1.3%) and 7 (2.3%) reported using EUCAST, BSAC and textbook respectively. Of the 154 not utilising AST reporting guidelines, 56 (18.5%) were not aware of AST guidelines, guidelines were not available in 67 (22.2%), 31(10.3%) use internal Standard Operating Procedure (SOP). Two hundred and twenty (72.8%) laboratories use qualitative method of reporting (intermediate and sensitive), 29 (9.6%) use both qualitative and quantitative (diameter) methods, 53 (17.6%) reported none. 169 (56.0%) laboratories store results on logbooks, 99 (32.8%) use computer files, 34 (11.3%) do not store AST results. The average readiness score for the laboratories was 62.91%: 118 (39.1%) laboratories were not ready for AMR surveillance, 162 (53.6%) were fairly ready, only 22 (7.3%) laboratories were fully ready for AMR surveillance (Table 2).

**Table 2:** Distribution of responses to items related to laboratory readiness to participate in AMR Surveillance (*n*=302)

Items	Responses *n*=302		*n* (%)
Laboratory readiness to participate in AMR surveillance			
Does your laboratory perform AST?	Yes		253 (83.8)
	No		49 (16.2)
Does your laboratory forward samples to other Laboratories for AST?	Yes		29 (9.6)
	No		20 (6.6)
What Method do you utilise for AST in your laboratory?			
Use Manual Method (Agar dilution and broth microdilution)			13 (4.3)
Use Manual Method (Disk diffusion and broth dilution)			136 (45.0)
Use Manual Method (Disk diffusion and E-test)			56 (18.5)
Use both Manual Method and automated system			25 (8.3)
Use both Manual Method and molecular techniques			29 (9.6)
Use broth dilution			23 (7.6)
We do not perform AST			20 (6.6)
How many GLASS pathogens does your laboratory carry out identification and antimicrobial susceptibility testing for?			
One GLASS priority organism			5 (1.7)
Two-Three GLASS priority organism			7 (2.0)
Four-Five GLASS priority organism			25 (8.3)
> Five GLASS priority organism			224 (74.2)
Other (none)			41 (13.6)
Are you aware of any AST guidelines?	Yes		183 (60.6)
	No		119 (39.4)
Does your laboratory utilise any AST guidelines?	Yes		128 (42.4)
	No		174 (57.6)
If Yes, what type?			
CLSI			95 (31.5)
EUCAST			22 (7.3)
BSAC			4 (1.3)
Textbook			7 (2.3)
If No, Why?			
Guideline not Available			67 (22.2)
Not aware of Any Guideline			56 (18.5)
Use internal SOP			31 (10.3)
We do not do AST			20 (6.6)
Method of Reporting AST Results			
Qualitative (R, I, S) and Quantitative (Diameter)			29 (9.6)
Qualitative (R, I, S)			220 (72.8)
None			53 (17.6)
Methods of Storing AST results			
Logbooks			169 (56.0)
Computer files			99 (32.8)
We do not store AST results			34 (11.3)
**Laboratory Readiness (%)**		M= 62.91 SD=29.02	
Not Ready			118 (39.1)
Fairly Ready			162 (53.6)
Fully Ready			22 (7.3)

Key: n=number, %=percentage, M=mean, SD=standard deviation

### Laboratory participation

Laboratory participation indicator was comprised of eight questions. Only 37 (12.3%) laboratories reported participation in AMR surveillance (Table 3). Two hundred and sixty-two (86.8%) laboratories do not submit data to any network. Of these 262 laboratories, 159 (52.9%) reported their facilities have not been listed to participate in AMR surveillance, 103 (34.1%) reported lack of personnel and infrastructure. Only 94 (31.1%) laboratories indicated AST results are always reviewed by senior technical staff or medical microbiologist before sending off the result. The average score for laboratory participation in AMR surveillance was 18.32%, only 37 (12.3%) laboratories were participating in AMR surveillance (Table 3).

**Table 3:** Distribution of responses to items related to laboratory participation in AMR Surveillance (*n*=302)

Items		Responses *n*=302	*n* (%)
Laboratory Participation in AMR surveillance			
Is your Laboratory participating in AMR surveillance?		Yes	37 (12.3)
		No	265 (87.7)
If yes, Length of Participation in AMR surveillance			
< 1 year			23 (62.2)
1-3 years			4 (10.8)
4-5 years			3 (8.1)
>5 years			7 (18.9)
Does your Laboratory report AST data to any Ministry, Organization or Surveillance network?		Yes	40 (13.2)
		No	262 (86.8)
If No, what are the significant obstacles faced by your laboratory in getting its data to any ministry or network?			
Our laboratory has not been invited to submit data			159 (52.6)
We need more equipment and personnel			103 (34.1%)
How often do you submit AST data?			
Monthly			3 (1.0)
Quarterly			19 (6.2)
Annually			18 (6.0)
We do not submit any report			262 (86.8)
Does your laboratory have internal SOP for assuring the quality of AST?		Yes	156 (51.7)
		No	146 (48.3)
Are all your tests reviewed before results are sent?		Yes	94 (31.1)
		No	208 (68.9)
If Yes, who reviews the result?			
Another member of the technical staff			56 (18.5)
A supervisor/medical microbiology			38 (12.6)
**Laboratory Participation (%)**	M= 18.32 SD= 19.66		
Not Participating			265 (87.7)
Participating			37 (12.3)

Key: n=number, %=percentage, M=mean, SD=standard deviation

### Recording of appropriate data

Recording of appropriate data for AMR surveillance comprised of items which helped to assess patients’ data collection, correlation of routine test results at the laboratories and compliance with WHO standards. One hundred and eighteen (39.1%) laboratories link AST result to all patient information (specimen source, patient bio-data, patient population…etc.), 132 (43.7%) laboratories link AST result to specimen source and patient bio-data only, 13 (4.3%) laboratories link AST results with clinical outcome, 39 (12.9%) laboratories do not link AST results to patients’ data. The criteria for recording all or select patients information vary across laboratories, 5 (1.7%) laboratories record patient population, pathogen type and infection origin only if the pathogen is generally considered clinically significant, 119 (39.4%) laboratories record patient population, pathogen type and infection origin only if the organism is considered clinically significant in the individual patient, 158 (52.3%) laboratories record patient population, pathogen type and infection origin from all isolates regardless of significant level of organism. Only 40 (13.2%) laboratories utilise WHONET for AMR data capturing. Four (1.3%) laboratories are reporting surveillance data to GLASS platform. The average score for recording of appropriate data for AMR surveillance was 30.35%. Only 3 (1.0%) laboratories record important AMR surveillance data, 107 (35.6%) partially record important AMR surveillance data, while 192 (63.6%) laboratories do not record important AMR surveillance data (Table 4).

**Table 4:** Distribution of responses to items related to appropriate recording of AMR Surveillance data (*n*=302)

Items	Responses *n*=302	*n* (%)
Are susceptibility results linked to any/all of the following data categories?		
AST results are linked to All Patient Information		118 (39.1)
AST results are linked to only Sample and Patient Bio-data		132 (43.7)
AST results are linked to clinical outcome		13 (4.3)
We do not link results to patient data		39 (12.9)
On what basis are patient population, Pathogen type and infection origin specifically recorded?		
Organisms generally considered clinically significant organisms		5 (1.7)
Organism considered clinically significant in individual patient		119 (39.4)
All organisms		158 (52.3)
We do not perform AST		20 (6.6)
Are guidelines established for the number and type of antibiotics resistance reported for organisms isolated from different sites of infection?	Yes	156 (51.7)
	No	146 (48.3)
Does your Laboratory use WHONET?	Yes	40 (13.2)
	No	263 (86.8)
Does your Laboratory report AST data to GLASS?	Yes	4 (1.3)
	No	298 (98.7)
Has your laboratory received any training on AMR in the last 3 years?	Yes	26 (8.6)
	No	276 (91.4)
**Recording of important AMR surveillance data (%)**	M= 30.35 SD= 24.65	
Not capturing AMR surveillance data		192 (63.6)
Partially capturing AMR surveillance data		107 (35.4)
Fully capturing AMR surveillance data		3 (1.0)

Key: n=number, %=percentage, M=mean, SD=standard deviation

### Surveillance quality indicator scores by laboratory affiliation (teaching, general, independent, FMCs, PHC, and private hospital)

A One-way ANOVA results indicated a statistically significant difference in mean knowledge score between respondents of the various groups of laboratory affiliation (F (5,297) = 14.29, *p* < 0.001). Similarly, there were statistically significant differences in the mean score of capacity (F (5, 297) = 66,38, *p* < 0.001), participation in AMR surveillance (F(5, 297) = 70.14, *p* < 0.001), readiness to undertake AMR surveillance (F(5, 297) = 12.47, *p* < 0.001) and recording of appropriate AMR data (F(5, 297) = 29.20, *p* < 0.001) between the various groups of laboratory affiliation. Overall, laboratories affiliated to teaching hospitals had better average scores from the post-hoc test.

### Association of demographic characteristics with knowledge and capacity

A lower odd of (OR = 0.72, 95%CI [0.52, 0.98]) knowledge was found among respondents from privately owned laboratories compared to their government. In terms of laboratory affiliation, there is a greater odd of better knowledge of AMR surveillance amongst laboratories affiliated to teaching hospitals compared to federal medical centre at an odd ratio of (OR = 2.35, 95%CI[1.45, 4.42], *p* = 0.008); similar trends were observed when the knowledge of laboratories affiliated with teaching hospital was compared with general/district hospital respondents at an odd ratio of (OR = 3.02, 95%CI [1.68, 5.43], *p* < 0.001); likewise a higher odd of (OR = 1.15, 95%CI (0.53–2.48), *p* < 0.001) was found when compared to laboratories affiliated with primary healthcare; (OR = 1.88, 95%CI [1.07, 3.31], *p* = 0.03) for laboratories affiliated to private hospital and (OR = 1.32, 95%CI [072, 2.45], *p* = 0.37) for laboratories affiliated to independent laboratories. A lower odd of (OR = 0.41, 95%CI [0.31, 0.56]) capacity was found amongst privately owned laboratories compared to the government which was used as the reference category (Supplementary Table 1).

### Association of demographic characteristics with readiness and participation

A lower odd of (OR = 1.07 95%CI 0.81–1.26) readiness was found amongst privately owned laboratories compared to government. A lower readiness was also found amongst laboratories affiliated to teaching hospitals (the reference category) compared to others. Laboratory participation was also significantly associated with laboratory ownership: government-owned laboratories are 59% (1-0.41) more likely to participate in AMR surveillance than privately owned. Also, laboratories affiliated to teaching hospitals were more likely to participate in surveillance than other laboratory affiliations (Supplementary Table 1).

### Correlation between knowledge, capacity, participation, readiness and data collection

A moderate positive correlation between laboratory capacity and participation [r (302) = 0.66, *p* = 0.001] was seen, as well as capturing important AMR data and laboratory participation [r (302) = 0.67, *p* = 0.001]. There was generally a positive correlation from one SQI to another, which shows that the performance of one SQI positively impacts the other, though at varying degrees.

## Discussion

This study investigated the laboratory capacity, capabilities and readiness for surveillance of AMR in Nigeria using a cross-sectional study approach and utilising surveillance quality indicators. This study reinforced the role that the laboratory plays in early detection of resistant pathogens crucial in tackling and managing the challenge of AMR [28]. Equally important for efficient and timely availability of data, is a systematic organisation of laboratories as highlighted in the report by [29], which observed that the participatory role of laboratories are enhanced when they are organised systematically within a system. This study was successful in gathering evidence from a large number of laboratories with wide coverage across the national and a diverse demographic across the six geo-political zones with respondents from laboratories in 35 of the 36 States and the FCT. As illustrated in Fig. 1, the Nigerian health system operates as a three-tiered structure managed by local, state, and federal governments. While this structure provides a broad network of facilities, fragmentation and resource disparities presents some barriers for AMR surveillance which is often concentrated in well-resourced tertiary centres. Despite these barriers, the system offers opportunities to strengthen AMR surveillance by leveraging the extensive reach of primary health centres, private laboratories, and improving coordination across all tiers to create a unified and more inclusive surveillance network.

Evidence from this study reveals absence of an organisational structures for collecting surveillance data at local, regional and national levels which impacts flow of surveillance data across time and space. There is ongoing routine surveillance but participation is disproportionately skewed towards tertiary care. The tertiary care is the highest referral level in the Nigerian healthcare organisational structure, and what this means for surveillance focused on tertiary care is that AMR in the population of people who access other levels of healthcare will not have any chance of being recorded. This could potentially be overestimating cases of AMR cases and consequently impact the representativeness of data.

Of particular concern is the absence of surveillance at the primary healthcare level. A large number of the population accessing primary care live in rural areas where potential for misuse of antimicrobials is high. Studies show high rates of misuse of antimicrobial agents amongst individuals in the rural settings due to over-the-counter purchase and absence of regulation on non-prescription access to drugs [30, 31, 32]. The organisational structure of the Nigerian healthcare system mirrors the governance structure of top-bottom approach with the lower tiers being prioritised the least. Thus, effective surveillance must take strategic approaches to ensure inclusion of the lowest level of healthcare as resistant infections do not respect geographical boundaries [33]. It is noteworthy that the burden of AMR is better estimated when surveillance is comprehensive rather than fragmented and until this happens, AMR estimates will remain largely exaggerated [34].

This study also assessed opportunities for increasing laboratory networks as well as improving quality of surveillance data. To determine this, laboratories were assessed on five SQIs (knowledge, laboratory capacity to undertake surveillance, readiness of the laboratory to participate in surveillance, status of laboratory participation in surveillance and capturing important surveillance data). As seen from the study outcome, there were significant differences between laboratory demographics (laboratory ownership, laboratory affiliation) and the SQIs. These have implications for future laboratory iterations into the surveillance system.

The knowledge indicator shows only 55 laboratories had excellent knowledge of AMR surveillance and the scores tapered down the laboratory hierarchy ladder with higher knowledge found amongst tertiary level laboratories. This finding correlates with the top-bottom structure earlier described. Despite efforts of the national coordinating centre (NCDC) to bridge this gap, including development of AMR surveillance guideline for laboratories in the country, the reach is still poor [35]. Knowledge is an important indicator for AMR containment which has been found to positively impact antimicrobial usage amongst healthcare providers and consumers [36]. An earlier study of antimicrobial use and resistance knowledge in the Nigerian population showed that knowledge of AMR was below 50% [37, 38]. These findings support other reports [39, 40] which demonstrated association between low knowledge of AMR and antimicrobial overuse. There were increases in antimicrobial use and resistance in places where AMR knowledge was low particularly in low and medium countries as exemplified in these studies thus, highlighting the knowledge pillar as crucial in AMR containment strategies. Government investment in knowledge needs to be all encompassing involving all categories of healthcare, and evidence from this study has not reflected that this is happening. Even at the laboratory level, only 26 (8.6%) laboratories reported to have undertaken training/continuing education in relation to AMR in the past three years and that does not reflect sufficient investment in clinician education.

The capacity assessment identified laboratories with potential for participating in national or early warning surveillance. These laboratories ranked at the same performance level as those currently participating in surveillance according to the study indicators. The most important requirements for participating in surveillance such as EQA enrolment and AST testing utilising the GLASS recommended disc diffusion methods were detected in some laboratories that were not involved in surveillance. This finding highlights the need to develop pathways to integrate under-utilised laboratories into the existing surveillance system in order to expand surveillance and increase representativeness. Other indicators of capacity for AMR surveillance such as use of reporting guidelines, accuracy checks, technical level of staff and equipment maintenance were equally assessed. These parameters help to assure the quality of laboratory testing procedure as well as ensure errors are eliminated from results through accuracy checks. Although the findings show that most technical staff were trained to conduct AST, procurement and maintenance of AST material and equipment were identified as a limitation factor. Only few laboratories participate in external quality assurance, with public sector laboratories’ having strong involvement in internal quality assurance programs. This gap in EQA participation between public and private sector laboratories may be impacting recruitment from private sector laboratories. To bridge this gap, mentoring of low baseline laboratories through continuous trainings have been advocated, including formulation and implementation of SOPs, frequent use of standardised quality control strains, and uniform inoculum for AST [41]. Studies from resource-limited countries have also shown efficacy of proficiency testing (PT) training programs in building sustainable network for knowledge and talent transfer through laboratory cooperation [4, 9, 15]. Such laboratory partnerships may reduce costs and increase diagnostic capabilities, thus providing a strong system for AMR surveillance.

Readiness was highest amongst government owned laboratories which corroborates several reports indicating that healthcare strengthening intervention disproportionately focus on government owned settings. Typical of this is the Fleming fund which was aimed at strengthening AMR capacity and laboratory upgrade but the beneficiaries were mostly tertiary government hospitals [42]. Tertiary hospitals are in an advantaged position being funded and managed by federal government, collaborate with the national reference laboratory as well as serve as a training centre for medical students. This spotlight position justifies their preference and the high readiness score recorded from this group. To be efficient, AMR surveillance needs to be comprehensive, and incorporate broad range of laboratories for a wider reach. There is urgent need for the inclusion of private sector laboratories for targeted laboratory improvement projects, by doing so, the private laboratories will be building the eligibility required to be part of the national surveillance.

Laboratory affiliation is another strong impacting factor revealed from this study. Results indicated that laboratories affiliated to teaching hospitals were more relevant to AMR surveillance. This is agreeable as teaching hospitals are more prepared and supported by government, multinational organisations and donor agencies [43, 44]. Another factor that support their preparedness is that most teaching hospitals also serve as centers for surveillance of other diseases and illness. For instance, in Nigeria, the US President’s Emergency Plan for AIDS Relief (PEPFAR) which was set up to strengthen surveillance capacity for AIDS in select tertiary laboratories provide opportunities to support for other disease surveillance [45]. Even though PEPFAR was not originally commissioned for AMR related activities, AMR surveillance could leverage on existing infrastructure of this project for seamless and integrated operation. Some of these opportunities are often not available to secondary and primary healthcare centers which impacts on their capacity, readiness and meeting the selection criteria for surveillance [46, 47].

AMR surveillance in Nigeria faces significant challenges but shows promise for integration under the “One Health” approach. Although the scope of this study is AMR surveillance in human, the findings showed that 7 (2.3%) laboratories evaluate samples from animals, and 5 (1.7%) from environment (Table 1). This opportunity can be leveraged to consolidate surveillance in animal and environment which is still developing. As we progress towards integrated surveillance, it is pertinent to close resource gap, enhance laboratory capacity and strengthen cross-sector collaboration.

## Conclusion

AMR surveillance implementation varies across laboratories, settings and regions. Laboratory capacity improvement programmes are more focused on government laboratories and surveillance participation is skewed towards tertiary laboratories. This widening equity gap between government and private affiliated laboratories as well as rural and urban healthcare services does not serve the purpose of good surveillance. Absence of surveillance at the lower-level laboratory means data on AMR situation from these levels are not picked up and consequently, soaring AMR rates in the community without data to inform control measures. Interestingly, a number of the lower-level laboratories reported excellent capacity as the laboratories currently participating in AMR surveillance but remain largely under-utilised. This could be attributed to recruitment shortfalls, lack of systematic laboratory assessment metrics and failure of oversight function by responsible bodies. These gaps have implications on representativeness and validity of surveillance data although findings from this study have highlighted ways of mitigating this problem.

### Limitation

The study assessed laboratory capacity using indicators such as reporting guidelines, accuracy checks, technical levels, and equipment maintenance. However, critical infrastructure (e.g., electricity and water), particularly relevant in LMICs, were excluded. These omissions may have influenced the capacity findings. This study utilised purposive sampling which has potential for selection bias, however steps were taken to minimise its occurrence, there are chances the recruitment approach might have introduced some bias which limits generalisability of the findings. The reliance on Yes/No responses to evaluate laboratory readiness for AMR surveillance may have overstated capacity and true compliance with AST guidelines. Results from this study relied on personal reports which are often characterised by intrinsic limitation such as exaggerated responses, underreporting weakness or overestimating strength. Although the authenticity of respondents were verified, they could be subject to self-reporting bias. Lastly, arbitrary ranking scores assigned to some SQIs might have led to inflated performance benchmarks. Despite these limitations, the study offers valuable insights into SQI scores, technical capacities, and vulnerabilities within various laboratory structures.

### Recommendation for further study

This study assessed the need for equipment among laboratories participating in AMR surveillance, further study is required to specifically investigate which pieces of key equipment are needed and their associated economic implication. These are useful indicators for planning expansion of surveillance networks and iteration.

## Electronic supplementary material

Below is the link to the electronic supplementary material.

Supplementary Material 1

## Data Availability

No datasets were generated or analysed during the current study.
