# Supplementary material for: Assessing barriers and opportunities for the improvement of laboratory performance and robust surveillance of antimicrobial resistance in Nigeria– a quantitative study
Source: Antimicrob Resist Infect Control. 2025 Apr 12;14:29. doi: 10.1186/s13756-025-01530-9 (PMC11993948; doi:10.1186/s13756-025-01530-9)
Supplement: Supplementary file 1 — Supplementary Material 1 [file 13756_2025_1530_MOESM1_ESM.docx]

Table 1 (supplementary data). **Association of demographic characteristics of laboratories in relation to knowledge of AMR surveillance, Laboratory Capacity scores, readiness for AMR surveillance and Laboratory participation scores (n=302)**

|  | **Knowledge** | | | **Lab Capacity** | | | **AMR Surveillance Readiness** | | | **Lab Participation** | | |
| --- | --- | --- | --- | --- | --- | --- | --- | --- | --- | --- | --- | --- |
|  | OR | 95%CI | p-value | OR | 95%CI | p-value | OR | 95%CI | p-value | OR | 95%CI | p-value |
| **Affiliation of Laboratory*** |  |  |  |  |  |  |  |  |  |  |  |  |
| Government Owned (Reference) |  |  |  |  |  |  |  |  |  |  |  |  |
| Private Owned | 0.72 | 0.52-0.98 | 0.04 | 0.41 | 0.31-0.56 | 0.0001 | 1.07 | 0.81-1.26 | 0.0001 | 0.41 | 0.25-0.67 | 0.0001 |
| **Level of Laboratory **** |  |  |  |  |  |  |  |  |  |  |  |  |
| Teaching Hospital (Reference) |  |  |  |  |  |  |  |  |  |  |  |  |
| Federal Medical Centre | 2.35 | 1.45-4.42 | 0.008 | 0.93 | 0.54-1.58 | 0.77 | 0.5 | 0.32-0.79 | 0.003 | 0.37 | 0.18-0.78 | 0.009 |
| General/District Hospital | 3.02 | 1.68-5.43 | 0.0001 | 1.15 | 0.69-1.88 | 0.35 | 0.45 | 0.29-0.69 | 0.0001 | 0.35 | 0.17-0.69 | 0.002 |
| Primary Health Care | 1.15 | 0.53-2.48 | 0.73 | 0.58 | 0.29-1.13 | 0.11 | 0.46 | 0.28-0.75 | 0.002 | 0.25 | 0.08-0.80 | 0.02 |
| Private Hospital Laboratory | 1.88 | 1.07-3.31 | 0.03 | 0.49 | 0.30-0.79 | 0.004 | 0.46 | 0.29-0.71 | 0.0001 | 0.28 | 0.13-0.58 | 0.001 |
| Independent Laboratory | 1.32 | 072-2.45 | 0.37 | 0.26 | 0.15-0.47 | 0.0001 | 0.53 | 0.34-0.84 | 0.006 | 0.05 | 0.02-0.12 | 0.0001 |
| **Geopolitical Zone of respondent laboratory**** |  |  |  |  |  |  |  |  |  |  |  |  |
| South-West (Reference) |  |  |  |  |  |  |  |  |  |  |  |  |
| South-South | 0.62 | 0.43-0.88 | 0.008 | 1.08 | 0.77-1.49 | 0.68 | 0.94 | 0.77-1.13 | 0.49 | 0.77 | 0.47-1.25 | 0.29 |
| South-East | 0.56 | 0.39-0.81 | 0.002 | 0.86 | 0.62-1.21 | 0.83 | 0.98 | 0.80-1.19 | 0.85 | 1.01 | 0.63-1.63 | 0.97 |
| North-Central | 0.86 | 0.60-1.23 | 0.4 | 0.32 | 0.89-0.64 | 0.39 | 0.84 | 0.69-1.02 | 0.08 | 0.88 | 0.55-1.41 | 0.59 |
| North-West | 0.48 | 0.28-0.82 | 0.007 | 1.49 | 0.94-2.36 | 0.09 | 0.79 | 0.62-1.01 | 0.06 | 1.38 | 0.68-2.82 | 0.37 |
| North-East | 0.38 | 0.22-0.66 | 0.001 | 1.13 | 0.71-1.82 | 0.6 | 0.77 | 0.61-0.98 | 0.03 | 1.49 | 0.73-3.07 | 0.27 |

Key: *=binary logistic regression, **=multinomial logistic regression, OR=odd ratio, 95%CI= 95% Confidence interval, p-value=Significant level, p<0.05=significant statistically, p>0.05=not significant statistically.
